# Impact of Antiretroviral Therapy on the Risk of Recurrence in HIV-1 Infected Patients with Kaposi Sarcoma: A Multicenter Cohort Experience

**DOI:** 10.3390/jcm8122062

**Published:** 2019-11-23

**Authors:** Manuela Colafigli, Arturo Ciccullo, Alberto Borghetti, Iuri Fanti, Federico Melis, Sara Modica, Ilaria Uccella, Antonio Bonadies, Virginia Ferraresi, Enza Anzalone, Alfredo Pennica, Emilia Migliano, Barbara Rossetti, Giordano Madeddu, Roberto Cauda, Antonio Cristaudo, Simona Di Giambenedetto, Alessandra Latini

**Affiliations:** 1 Infectious Dermatology and Allergology, San Gallicano Dermatological Institute IRCCS, 00144 Rome, Italy; 2 Clinical Infectious Diseases, Catholic University of S. Heart, 00168 Rome, Italy; 3 Unit of Infectious Diseases, Department of Medical, Surgical and Experimental Sciences, University of Sassari, 07100 Sassari Italy; 4 Department of Medical Biotechnologies, Azienda Ospedaliera Universitaria Senese, 53100 Siena, Italy; 5 Infectious Diseases Unit, Azienda Ospedaliera Universitaria Senese, 53100 Siena, Italy; 6 UOC Malattie Infettive, 03100 Frosinone, Italy; 7 Plastic Surgery, San Gallicano Dermatological Institute IRCCS, 00144 Rome, Italy; 8 First Division of Medical Oncology, IRCCS Regina Elena National Cancer Institute, 00144 Rome, Italy; 9 UOSD AIDS, 03100 Frosinone, Italy; 10 AOU S Andrea, 00189 Rome, Italy

**Keywords:** HIV infection, acquired immunodeficiency syndrome (AIDS), Kaposi sarcoma

## Abstract

Kaposi sarcoma (KS) remains a relevant malignancy in human immunodeficiency virus (HIV)-infected patients with a non-standardized management; despite past suggestions that ritonavir-boosted protease inhibitor (bPI)-based regimens could be preferable, no combination antiretroviral therapy (cART) regimen was demonstrated to outperform the others and the impact of new drugs, drug classes or paradigms was never investigated nor proven better than previous therapeutic regimes. In order to do this, we retrospectively collected data regarding HIV-infected patients with a diagnosis of KS last seen in six Italian centers after 1 January 2013. A total of 104 KS cases in 99 patients was analyzed for 945.34 patient-year follow-up (PYFU). Twenty-six patients had visceral localizations. Thirty-three patients were treated with chemotherapy, four with electrochemotherapy, and 12 with α-interferon (α-IFN). At censor, 22% received a bPI-based, 14% a non-nucleoside reverse transcriptase inhibitor (NNRTI)-based, and 28% an integrase inhibitor (INI)-based standard cART, 24% a less drug regimen and 12% a mega-cART. Twelve recurrence episodes were observed in seven patients for an incidence of 1.27 per 100 PYFU. Two patients with no evidence of recurrence episodes died for other reasons. In our experience, KS recurrence episodes were infrequent. Despite the increasing use of new antiretroviral drug classes and new treatment paradigms, no excess of recurrence episodes was observed in patients receiving such cART regimens.

## 1. Introduction

In more than thirty years since acquired immunodeficiency syndrome (AIDS) was defined, the introduction and the subsequent optimization of combination antiretroviral therapy (cART) greatly improved its prognosis [1] and the incidence of many associated events, including Kaposi sarcoma (KS), declined over the years [2,3]. Nevertheless, the incidence of KS remains relevant, particularly among men who have sex with men (MSM) [4], and it can still be frequently seen as a late presentation or in the context of immune reconstitution syndrome (IRIS) [5,6] and even, although less frequently, in patients with stable viroimmunological success [7,8,9]. International guidelines recognize cART as the only possible therapy for patients with initial, mild to moderate cutaneous KS disease [10,11,12,13], adding chemotherapy in the context of IRIS of rapidly progressive diseases or of visceral localizations [13]. 

Therefore, when KS is diagnosed, international guidelines recommend the rapid initiation of cART, with or without chemotherapy if indicated [10,11,12,13]. Nevertheless, there are no specific indications concerning the best cART regimen in this situation. In the early ART era, a possible advantage on KS with the use of protease inhibitors (PIs) and ritonavir was found in some studies [14,15,16,17,18]; although no definitive evidence was ever provided, PIs were often the first choice for the initiation of cART in human immunodeficiency virus (HIV)-infected patients with KS. Finally, sporadic cases of recurrence of KS in patients switching away from protease inhibitors are reported in the literature [19,20]. On the contrary, a large cohort study denied an advantage of PIs over non-nucleoside reverse transcriptase inhibitors (NNRTIs) [21] and the development of KS was also reported in patients who were successfully treated with boosted PIs [7].

Currently, major guidelines recommend the use of integrase inhibitors (INIs) as first line cART in the vast majority of cases [10,11,12,13], so the use of this class has increased all around the world and in many settings, including patients with KS. In addition, cobicistat as a booster has widely replaced ritonavir among PIs. Moreover, the use of less drug regimen (LDR, defined as dual regimens built with a high genetic barrier antiretroviral such as a boosted PI or dolutegravir plus a second antiretroviral drug or, less frequently, a boosted PI monotherapy, similar to the class-sparing strategies defined in the EACS guidelines [12]) has become increasingly common as an optimization regimen in patients with viroimmunological success as a possibility to avoid nucleos(t)ide reverse transcriptase inhibitor (NRTI)-related toxicity after various studies confirmed their value in switch strategies [22,23,24,25,26,27,28]. The use of this strategy could further increase following the success of the more recent dolutegravir-based LDR studies in both naïve and treatment-experienced populations [27,28,29]. However, no specific data are available on the efficacy and safety of such strategies in HIV-infected patients with KS.

The aim of this study was to evaluate the incidence of recurrence episodes (REs) and the possible associations with the prescribed antiretroviral regimens.

## 2. Experimental Section

This was a retrospective, observational, multicentric study conducted in six Italian centers. Clinical and laboratory data regarding all HIV-infected patients last seen in our outpatient services after 01 January 2013 were collected; clinical history including prior exposure to cART, exposure to chemotherapy (CT), electrochemotherapy (ECT) or α-interferon (α-IFN) were also recorded. Baseline was set at the date of the diagnosis of KS, follow-up was censored at the first recurrence episode (RE) or at the last observation or 15 March 2019. A RE was defined as the appearance of a new KS lesion after prior clinical remission.

HIV testing was performed by ELISA assay with Western blot confirmation. KS diagnosis was defined by skin or mucosal biopsy; all patients diagnosed with KS were staged at baseline (esophagogastroduodenoscopy, colonoscopy, and total body CT scan).

Liposomal doxorubicin was used for chemotherapy at the recommended dose of 20 mg/m^2^ every three weeks [30]. Intravenous bleomycin was administrated intravenously eight minutes before electrochemotherapy as previously described [31]. Alpha-interferon was administered at the dose of 6,000,000 units with a subcutaneous injection every other day.

Descriptive analysis was performed as appropriate for continuous and categorical variables; survival free from recurrence episode was estimated by Kaplan–Meier (KM) analysis. Patients with multiple REs were evaluated as a single case for descriptive statistical analysis regarding demographic characteristics and as many cases as the number of REs for the KM-estimated survival free from recurrences and descriptive analysis regarding the exposure to antiretroviral drugs. The incidence of recurrence episodes was calculated as the number of events during the total follow-up time.

## 3. Results

### 3.1. Study Population

A total of 99 patients from six centers were enrolled. Table 1 shows the main demographic characteristics for the whole study population at baseline and stratified by those with and those without recurrence episodes. 

### 3.2. KS Clinical Details and Outcome

A skin or mucosal biopsy was available for all patients at baseline and at the recurrence episodes. In 26 patients, a visceral localization was found in one or more sites: 18 out of the 26 (69.2%) had oral cavity mucosal lesions, 9 (34.6) had gastric lesions, 6 (23%) had colic lesions, and 8 (30.8%) had pulmonary lesions.

In seven patients the KS lesions were completely removed by biopsy; four out of the seven patients underwent chemotherapy thereafter and one of them also underwent electrochemotherapy. A total of 33 (33.3%) patients were treated with a median (interquartile range (IQR), range) of six (4–9; 2–52) cycles of chemotherapy for a median (IQR) time of 5.2 (2.6–12.6) months, 4 (4%) with electrochemotherapy, and 12 (12%) with α-IFN for a median (IQR, range) time of 14.2 (6.8–41.4; 1.7–58.3) months. The remaining patients were managed with antiretroviral therapy alone.

Table 2 shows the clinical details of KS and outcome stratified by patients with or without recurrence episodes.

The disappearance of the cutaneous lesions was well documented in the clinical records for 62 (62.6%) patients; visceral remission was documented for all 26 patients with initial visceral localizations.

Twelve recurrence episodes were observed in seven patients, four of which had multiple episodes (3/4 had two and 1/4 had three recurrence episodes), while the remaining three had just one recurrence episode, for an incidence of 1.27 per 100 PYFU. Median (IQR) time free from new episodes was 7.48 (3.19–14.05) years. Table 3 shows the clinical details of the 12 cases with recurrence episodes, including the temporal relationship between the time of KS and HIV diagnosis, time of cART initiation, and date of recurrence event. All patients were virologically suppressed at the time of the event. 

Patient no. 4 was the only HIV-negative patient at the diagnosis of KS. The diagnosis was made elsewhere in July 2011 (age 47), when the biopsy of an oral cavity localization revealed KS; a few months earlier, other KS lesions of the left leg and foot had appeared. He first came to our observation on 26 March 2013 bringing negative HIV tests; CD4 cell count was 569 cells/mmc (30%, CD4/CD8 ratio 0.74), HHV8 PCR was positive but lower than 125 genome equivalents/mL. He underwent ECT in May. He was tested for HIV infection on 19 July 2013 with negative results; CD4 cell count was 420 cells/mmc (35%, CD4/CD8 ratio 1.19) and HHV8 PCR was 284 genome equivalents/mL. He was again tested negative for HIV infection a third time on 24 October 2013, when a new KS lesion was found in his back; HHV8 PCR was positive but lower than 125 genome equivalents/mL and CD4 cell count was 389 cells/mmc (39%, CD4/CD8 ratio 1.16). Finally, he tested positive for HIV (index: 23, viral load 463,064 copies/mL) on the subsequent visit on 17 December 2013; HHV8 PCR was stably positive but lower than 125 genome equivalents/mL and CD4 cell count 329/mmc (22%, CD4/CD8 ratio 0.39). We prescribed him a mega-cART regimen as described in Table 3 and he reached virologic suppression in July 2014 (CD4 cell count 441/mmc, 31%, CD4/CD8 ratio 0.69); nevertheless, new KS lesions were detected. He also underwent 20 ECTs and 52 CT cycles with liposomal doxorubicin in 29.4 months and, after a final recurrence episode in October 2015 (CD4 cell count 392/mmc, 36%, CD4/CD8 ratio 0.93, HIV RNA <40 copies/mL), clinical remission has been observed since 30 June 2017. 

Patient no. 5 was diagnosed with HIV infection in 1992 (age 45). He was exposed to suboptimal NRTI-only therapy until he started an unboosted PI-based regimen in 1998 as described in Table 3. He changed 10 cART regimens due to toxicity (peripheral neuropathy and dyslipidemia) and as he grew older, he developed increasingly severe muscular dystrophy, so his cART was switched to a NNRTI-based regimen (2007–2015) and then to the LDR lamivudine plus ritonavir-boosted atazanavir regimen (March 2015) during these changes. At the time, his CD4 cell count was 462 cells/mmc (20%, CD4/CD8 ratio 0.5) and his HIV viral load was undetectable (target not detected with real time (RT) PCR Taqman assay). Nevertheless, a KS lesion was detected, and the diagnosis was confirmed by biopsy; the genetic barrier of his cART regimen was increased by switching to dolutegravir plus ritonavir-boosted atazanavir. Despite persistently undetectable HIV viral load and a CD4 cell count stably higher than 450 cells/mmc, we observed two recurrence episodes as described in Table 3. In January 2019 he was switched to a tenofovir alafenamide/emtricitabine plus dolutegravir standard three drug regimen with good tolerability and no further recurrence episodes at censor.

Patient no. 7 was diagnosed with HIV infection in 1995 elsewhere and came to our attention in 2004, following the appearance of cutaneous lesions compatible with KS, confirmed by biopsy. Staging for organ localizations resulted negative. The patient was still naïve to antiretroviral therapy. Despite the successful introduction of cART (CD4 1205/mmc, HIV viral load <50 copies/mL), we witnessed the appearance of new skin lesions; hence, the patient started six cycles of CT with liposomal doxorubicin, with benefit (inactive lesions). After two years, in 2008, new purple lesions appeared despite continued virologic success (CD4 1452/mmc, HIV viral load <50 copies/mL); no chemotherapy was started, and lesions disappeared with antiretroviral therapy alone. The patient is still in follow-up in our center, his cART was switched to an INI-based standard regimen (2012–2015), and then to the lamivudine plus dolutegravir LDR regimen with no further signs of KS and a stable virologic suppression.

**Figure 1:**
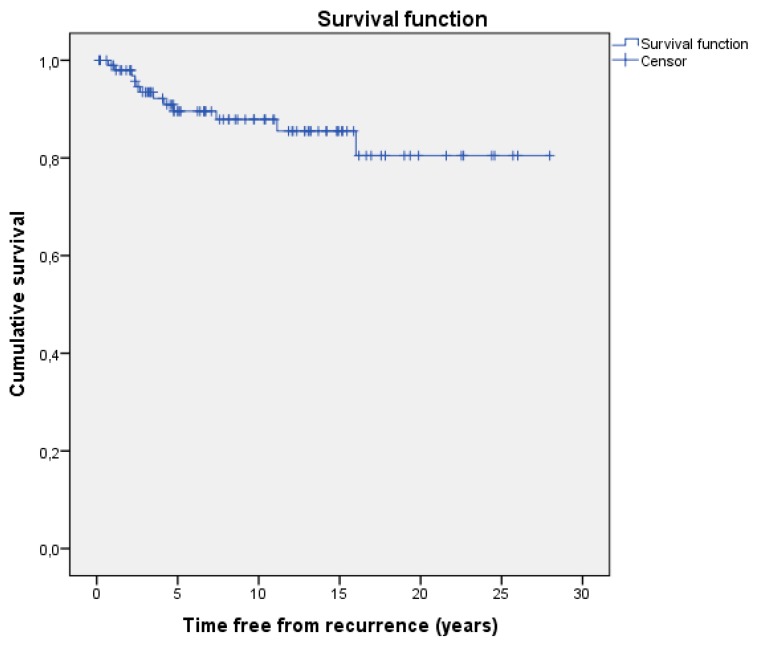
Kaplan–Meier estimated survival free from Recurrence Events.

Two deaths among the 99 patients were observed: one patient died because of a sepsis two months after the KS and HIV infection were diagnosed and one patient was lost to follow-up after the first month; he was subsequently reported as dead for unknown reasons by his family. For both patients, no evidence of recurrence of KS was available. Figure 1 shows the overall Kaplan–Meier estimated survival free from RE and Table 4 shows its risk table.

### 3.3. Antiretroviral Therapy

Table 5 and Table 6 show the exposure to different antiretroviral regimens of the patient population as a whole and stratified by recurrence episodes. In Table 5 we show the antiretroviral regimens prescribed at baseline and at censor, while in Table 6 we detail the cumulative exposure to the different antiretroviral drug classes and drugs. The very low number of cases in the group of patients with recurrences prevented us from evaluating their median time of exposure to the newest drugs and drug classes.

At censor, the proportion of patients receiving a boosted PI-based cART regimen had decreased while the proportion of patients receiving INI-based regimens, cobicistat-boosted regimens or LDR regimens had increased.

## 4. Discussion

KS remains a relevant AIDS-defining malignancy and it still represents the second most frequent AIDS-defining event among MSM in Italy [4] and the second most frequent AIDS-defining event among late presenters in another large Italian cohort [6]. The use of cART remains the only treatment for mild to moderate cutaneous diseases but there is no evidence about the best regimen for this setting and recent changes in the recommended first line regimens and possible future changes of paradigms for the treatment of HIV infection could have an impact on the control of this AIDS-defining event.

This study tried to describe the incidence and characteristics of KS recurrence in six major Italian centers; we choose to only insert patients who last accessed our services after 01 January 2013 in order to include patients who had the possibility to receive the newest and most recently recommended therapies. Therefore, the median calendar year of KS diagnosis was quite recent (2010, IQR 2004–2015), even if the range went from 1989 to 2018. The median exposure was slightly less than two years for the newest compounds such as INIs and dolutegravir in particular, and the cobicistat booster; in this limited exposure time, we did not find any excess of recurrences in the exposed patients.

The follow-up time on LDR exposure is slightly less than four years with a wide IQR and an upper limit above five years, reflecting the long time experience of our centers with this strategy; again, no excess of recurrences was observed in patients exposed to these regimens, with or without protease inhibitors and independently from the type of booster.

Recurrence episodes were infrequent. As indicated in the details shown in Table 3, however, no common ground could be identified in patients with one or more event. Moreover, the cART regimens that these patients were receiving were well diversified, including every kind of antiretroviral drug class, booster or treatment paradigm, so no real possible clinical disadvantage could be implied. Patient no. 4, the one with the most severe disease, was diagnosed with KS long before he acquired HIV infection; other such cases have been occasionally described in literature [32,33]. This patient immediately provided many difficulties in his management; nevertheless, after 52 chemotherapy cycles between October 2014 and March 2017, he is currently on stable remission since the end of June 2017. The patient was treated with a mega-cART regimen including boosted darunavir and raltegravir since his HIV infection was diagnosed.

Our study has several limitations. Firstly, it is an observational, retrospective study, so the data regarding clinical evolution and local and/or systemic therapies are often missing/incomplete and the virologic data regarding HHV8 is absent and therefore not evaluated in the majority of centers. Secondly, the follow-up time with new drugs/boosters/drug classes is limited, although almost two years seems to be a reasonable interval to try to drive some early observations. Finally, the old perception that PIs could have an advantage over other antiretroviral classes could have led to a prescription bias in the first cART regimens, but the increasing part of patients receiving new drug classes, namely integrase inhibitors, in more recent years proves that this effect is declining over time. Furthermore, the introduction of the less drug regimen did not seem to affect the control of both HIV and KS.

Nevertheless, this study has the advantage to be, to our knowledge, the first to investigate the impact of the newest cART regimens and paradigms on KS; in fact, only sporadic case reports about the effect of integrase inhibitors [19,20,34] are present in the literature. Further studies with longer follow up time in larger cohorts are warranted, but our data can probably provide a reasonable proof of concept that newer drug classes and new paradigms may be safe, especially in patients with long time KS remission.

In conclusion, in our experience KS recurrence events were infrequent. Despite the increasing use of new antiretroviral drug classes and new treatment paradigms, no excess of KS recurrence episodes was observed in patients receiving such cART regimens.

## Figures and Tables

**Table 1:** Patients’ demographic characteristics at baseline.

Variable	Whole Patient Population (*n* = 99)	Patients with No Recurrence Episodes (*n* = 92)	Patients with Recurrence Episodes (*n* = 7)
Male gender, *n* (%)	95 (95.9)	88 (95.7)	7 (100)
Age, years: median (IQR); range	41 (35–46); (26–70)	41 (36–46); (26–70)	40 (34–47); (31–67)
Risk factor, *n* (%)			
Heterosexual contact	17 (17.2)	17 (18.5)	
MSM	73 (73.7)	66 (71.7)	7 (100)
IDU	1 (1)	1 (1.1)	
Other/Unknown	8 (8.1)	8 (8.7)	
Caucasian ethnicity, *n* (%)	94 (94.9)	87 (96.7)	7 (100)
HCV coinfection, *n* (%)	1 (1.2)	1 (1.1)	0
HBV coinfection, *n* (%)	3 (3.6)	2 (2.6)	1 (14.3)
Year KS was diagnosed: median (IQR); range	2010 (2004–2015); (1989–2018)	2010 (2004–2015); (1989–2018)	2009 (2004–2011); (2000–2015)
Nadir CD4 cell count: median (IQR) cells/mmc	142 (56–252)	141 (56–242)	233 (61–362)
Zenith VL: median (IQR) log10 copies/mL	5.23 (4.77–5.57)	5.25 (4.78–5.59)	4.88 (4.68–5.55)
CD4 cell count at BL: median (IQR) cells/mmc			
Absolute CD4 cell count	141 (48–294)	140 (48–265)	318 (61–537)
CD4 cell count percent value	10 (4–19)	10 (4–15)	22 (5–26)
CD4/CD8 ratio	0.18 (0.08–0.37)	0.18 (0.08–0.31)	0.45 (1.59–5.55)
VL at BL: median (IQR) log10 copies/mL	5.13 (4.35–5.55)	5.14 (4.38–5.54)	4.74 (1.59–5.55)

IQR: interquartile range, MSM: men who have sex with men, IDU: intravenous drug use, KS: Kaposi sarcoma, BL: baseline, VL: HIV Viral Load.

**Table 2:** KS clinical details and outcome stratified by patients with or without recurrence episodes.

Variable	Patients with No Recurrence Episodes (*n* = 92)	Patients with Recurrence Episodes (*n* = 7)
Visceral localizations, *n* (%)	25 (28.1)	1 (14.3)
Oral mucosa	17 (19.5)	1 (14.3)
Gastric	9 (10.2)	0
Colic	6 (6.8)	0
Pulmonary	8 (9.0)	0
Completely removed by biopsy, *n* (%)	6 (6.7)	1 (14.3)
Chemotherapy, *n* (%)	30 (33.0)	3 (42.9)
Electrochemotherapy (ECT), *n* (%)	3 (3.3)	1 (14.3)
α-Interferon, *n* (%)	11 (12.0)	1 (14.3)

**Table 3:** Clinical details of the patients with one or more recurrence episode(s).

Patient ID	Date KS Was Diagnosed	Date HIV Infection Was Diagnosed	Date cART Was Started	1st Line cART Regimen	cART at censor	No. of REs	Date of RE
1	11/01/2002	17/01/2002	08/02/2002	AZT/3TC + IDV	3TC + ATVc	1	12/01/2018
2	27/09/2005	27/09/2005	08/10/2005	TDF/FTC/EFV	TDF/FTC/EFV	1	09/02/2013
3	03/11/2009	/08/2006	20/01/2007	TDF/FTC + LPVr	TDF/FTC + LPVr TDF/FTC + LPVr	2	23/07/2010 02/01/2012
4*	15/01/2011	/12/2013	29/01/2014	TDF/FTC + DRVc + RAL	None * TDF/FTC + DRVc + RAL TDF/FTC + DRVc + RAL	3	26/05/2013 18/07/2014 15/10/2015
5	03/03/2015	15/06/1992	26/06/1998	D4T + 3TC + IDV	3TC + ATVr DTG + ATVr	2	09/04/2016 07/11/2017
6	02/03/2000	18/01/2000	20/03/2000	AZT/3TC + IDV	TDF/FTC/EFV	1	21/04/2011
7	13/05/2004	12/08/1995	15/04/1998	D4T + DDI + SQV	TDF/FTC + ATVr TDF/FTC + ATVr	2	04/10/2006 14/07/2008

* Patient no. 4 still had not acquired HIV infection at the time of his first recurrence episode, therefore he was not receiving cART at that time. AZT: zidovudine, D4T: stavudine, DDI: didanosine, 3TC: lamivudine, FTC: emtricitabine, TDF: tenofovir disoproxil fumarate, EFV: efavirenz, IDV: indinavir, SQV: saquinavir, LPVr: lopinavir/ritonavir, ATVr: ritonavir-boosted atazanavir, ATVc: cobicistat-boosted atazanavir, DRVc: cobicistat-boosted darunavir, RAL: raltegravir, DTG: dolutegravir.

**Table 4:** Risk table for the Kaplan-Meier estimated survival free from Recurrence Events.

	1 Year	3 Years	5 Years	10 Years
Estimated survival (SD)	99 (0.010)	93.5 (0.026)	89.6 (0.033)	87.9 (0.037)
No. of cumulative events	1	6	9	10
No. of remaining cases	97	81	63	42

**Table 5:** Antiretroviral drug regimens and viroimmunological parameters at baseline and at censor.

Variable	Whole Patient Population (*n* = 104)	Cases with No Recurrence Episodes (*n* = 92)	Cases of Recurrence Episodes (*n* = 12)
Exposure to suboptimal ART, *n* (%)	46 (44.2)	41 (44.6)	5 (41.7)
Exposed to cART when KS was diagnosed, *n* (%)	36 (35)	30 (33)	6 (50)
Started cART after KS was diagnosed, *n* (%)	61 (65)	61 (67)	6 (50)
First cART regimen, *n* (%)			
Standard 3 drug bPI-based regimen, *n* (%)	50 (48.1)	48 (52.2)	2 (16.7)
Standard 3 drug NNRTI-based regimen, *n* (%)	6 (5.8)	5 (5.4)	1 (8.3)
Standard 3 drug INI-based regimen, *n* (%)	9 (8.7)	9 (9.8)	0
Dual NRTI regimens	9 (8.7)	9 (9.8)	0
2 NRTI + unboosted PI	19 (18.3)	13 (14.1)	6 (50)
Mega cART	11 (10.6)	8 (8.7)	3 (25)
cART at censor:			
Standard 3 drug bPI-based regimen, *n* (%)	22 (22)	18 (20.2)	4 (36.4)
Standard 3 drug NNRTI-based regimen, *n* (%)	14 (14)	12 (13.5)	2 (18.2)
Standard 3 drug INI-based regimen, *n* (%)	28 (28)	28 (31.5)	0
PI-based LDR, *n* (%)	17 (17)	14 (15.7)	3 (27.3)
Dolutegravir-based LDR, *n* (%)	7 (7)	7 (7.9)	0
Mega cART, *n* (%)	12 (12)	10 (11.2)	2 (18.2)
CD4 cell count at censor: median (IQR) cells/mmc	552 (412–811)	551 (399–811)	542 (446–804)
VL at censor: median (IQR) log10 copies/mL	1.59 (1.56–1.59)	1.59 (1.56–1.59)	1.59 (1.59–1.66)
Undetectable (target not detected), *n* (%)	48 (48)	45 (50.6)	3 (27.3)
Low level viremia (1–50 copies/mL), *n* (%)	46 (46)	38 (42.7)	8 (72.7)
Detectable (>50 copies/mL), *n* (%)	6 (6)	6 (6.7)	0

ART: antiretroviral therapy, cART: combination ART, KS: Kaposi sarcoma, NNRTI: non-nucleosidic reverse transcriptase inhibitors, PI: protease inhibitors, bPI: boosted protease inhibitors, INI: integrase inhibitors, LDR: less drug regimen.

**Table 6:** Cumulative exposure to antiretroviral drugs.

Variable	Whole Patient Population (*n* = 102)	Cases with No Recurrence Episodes (*n* = 90)	Cases of Recurrence Episodes (*n* = 12)
Ever exposed to unboosted PI-based regimens, *n* (%)	31 (29.8)	25 (27.2)	6 (50)
Cumulative time on uPI-based regimen, months: median (IQR)	41.2 (20.5–66.6)	49.7 (22.4–99.7)	24.4 (15.6–41.2)
Ever exposed to boosted PI-based regimens, *n* (%)	89 (85.6)	81 (88)	8 (66.7)
Cumulative time on bPI-based regimen, months: median (IQR)	67.5 (33.9–120.7)	65.2 (30.6–121.3)	89.2 (66–117)
Ever exposed to NNRTI-based regimens, *n* (%)	50 (48.1)	43 (46.7)	7 (58.3)
Cumulative time on NNRTI-based regimen, months: median (IQR)	57.4 (16.8–92.1)	42.1 (13.3–86.3)	108.9 (60.9–163.8)
Ever exposed to INI-based regimens, *n* (%)	54 (51.9)	51 (55.4)	3 (25)
Raltegravir	24 (23.1)	22 (23.9)	2 (16.7)
Elvitegravir	10 (9.6)	10 (10.9)	0
Dolutegravir	36 (34.6)	35 (38)	1 (8.3)
Cumulative time on INI-based regimen, months: median (IQR)	29.3 (16.4–46.1)	31.8 (16.4–47.4)	N.E.
Cumulative time on raltegravir, months: median (IQR)	20.6 (5.7–48.9)	28.2 (6.1–51.0)	N.E.
Cumulative time on elvitegravir, months: median (IQR)	26.9 (13.1–29.9)	26.9 (13.1–29.9)	N.E.
Cumulative time on dolutegravir, months: median (IQR)	21.2 (14.2–34.9)	21.2 (13.8–35.6)	N.E.
Ever exposed to cobicistat-boosted regimens, *n* (%)	48 (46.2)	47 (51.1)	1 (8.3)
Darunavir-cobicistat	27 (26)	27 (29.3)	0
Atazanavir-cobicistat	17 (16.3)	16 (17.4)	1 (8.3)
Cumulative time on cobicistat, months: median (IQR)	21.2 (10.2–23.7)	21.2 (10.3–23.8)	N.E.
Cumulative time on darunavir-cobicistat, months: median (IQR)	15.9 (6.0–22.6)	15.8 (5.8–22.2)	N.E.
Cumulative time on atazanavir-cobicistat, months: median (IQR)	21.0 (10.3–23.1)	21.6 (9.8–23.3)	N.E.
Ever exposed to LDR, *n* (%)	48 (46.2)	45 (48.9)	3 (25)
Cumulative time on LDR, months: median (IQR)	46.7 (14.7–76.4)	49.5 (14.7–79.8)	N.E..
Ever exposed to mega cART, *n* (%)	28 (26.9)	24 (26.1)	4 (33.3)
Cumulative time on Mega cART, months: median (IQR)	20.2 (5.6–43.4)	17.8 (5.0–33.6)	47.5 (9.5–74.3)
Time on specific cART regimens at censor:			
Boosted PI-based regimen, months: median (IQR)	17.3 (11.0–25.0)	16.5 (10.7–24.1)	20.8 (12–70.3)
NNRTI-based regimen, months: median (IQR)	14.6 (13.5–85.9)	14.4 (12.5–39.5)	N.E.
INI-based regimen, months: median (IQR)	19.9 (12.3–28.6)	19.8 (12.3–30.3)	N.E.
Raltegravir-based regimen, months: median (IQR)	15.5 (5.8–21.4)	15.5 (6.1–22.2)	N.E.
Elvitegravir-based regimen, months: median (IQR)	21.4 (12.8–33.6)	21.4 (12.8–33.6)	N.E.
Dolutegravir-based regimen, months: median (IQR)	22.2 (13.5–30.8)	22.2 (13.1–32.0)	N.E.
Cobicistat-boosted regimen, months: median (IQR)	15.3 (10.4–22.7)	15.3 (10.5–22.1)	N.E.
Darunavir/cobicistat-based regimen, months: median (IQR)	13.5 (8.4–17.5)	12.5 (7.4–17.3)	N.E.
Atazanavir/cobicistat-based regimen, months: median (IQR)	17.3 (10.3–23.8)	19.1 (11–24.1)	N.E.
LDR regimen, months: median (IQR)	18.1 (13.0–25.6)	18.4 (13.2–31.1)	N.E.
Mega cART, months: median (IQR)	18.6 (5.8–23.5)	18.2 (5.1–43.8)	N.E.

ART: antiretroviral therapy, cART: combination ART, KS: Kaposi sarcoma, NNRTI: non-nucleosidic reverse transcriptase inhibitors, PI: protease inhibitors, bPI: boosted protease inhibitors, INI: integrase inhibitors, LDR: less drug regimens, N.E.: not evaluated.
